# Storage and retrieval of deceased patients’ data: a bioethical reflection from a Kantian deontological perspective

**DOI:** 10.1590/0034-7167-2025-0302

**Published:** 2026-07-24

**Authors:** Bethânia Karoline Alvaro Menezes, Marcos Antonio Ferreira, Aline Moraes Silva, Andreia Insabralde Queiroz-Cardoso, Viviane Euzébia Pereira Santos

**Affiliations:** I Universidade Federal de Mato Grosso do Sul. Campo Grande, Mato Grosso do Sul, Brazil; II Universidade Federal do Rio Grande do Norte. Natal, Rio Grande do Norte, Brazil

**Keywords:** Information Storage and Retrieval, Death, Ethical Theory, Bioethics, Reflective Thinking., Almacenamiento y Recuperación de la Información, Muerte, Teoría Ética, Bioética, Pensamiento Crítico.

## Abstract

**Objectives:**

to examine the bioethical aspects of storing and retrieving deceased patients’ data for scientific research from a Kantian deontological perspective.

**Methods:**

theoretical reflection grounded in Kantian deontological ethics and national and international legislation.

**Results:**

there are significant gaps in the regulation of the use of deceased patients’ data in Brazil. National standards on research ethics and data protection do not clearly address this issue, whereas international models provide different solutions that take into account patients’ *ante-mortem* wishes regarding the *post-mortem* use of their data. Kantian deontology conceives of dignity as an absolute value that persists beyond biological life and requires that autonomy and patients’ previously expressed wishes be respected.

**Final Considerations:**

we suggest creating legal instruments for *ante mortem* consent to the *post-mortem* use of personal data, allowing patients to decide how their data will be used after death and safeguarding their dignity and autonomy.

## RESUMEN

**Objetivos:**

examinar los aspectos bioéticos del almacenamiento y la recuperación de datos de pacientes fallecidos para investigación científica desde la deontología kantiana.

**Métodos:**

ensayo teórico-reflexivo fundamentado en la ética deontológica kantiana y en normativas nacionales e internacionales.

**Resultados:**

se identifican lagunas en la regulación brasileña del uso *post mortem* de datos personales; las normas nacionales de ética en investigación y de protección de datos no abordan con claridad el tema, mientras que modelos internacionales ofrecen soluciones que consideran la voluntad expresada en vida. La deontología kantiana concibe la dignidad como valor absoluto que persiste más allá de la vida biológica y exige respeto a la autonomía y a la voluntad previamente expresada.

**Consideraciones Finales:**

se propone crear instrumentos jurídicos de consentimiento en vida que permitan a las personas decidir el uso *post mortem* de sus datos, para salvaguardar su dignidad y autonomía.

## INTRODUCTION

Death is understood in many different ways across cultures and religions, and the biological process of dying consists of a sequence of gradual phenomena rather than a single, finite moment^(1)^. This view has practical implications, particularly for scientific research that uses information about patients after their death.

Advances in health research increasingly depend on access to detailed, accurate information, often including sensitive data from deceased patients. The collection, storage, and analysis of such information raise important ethical issues because, from a Kantian deontological perspective, respect for human dignity is an absolute value that does not end with death^(2,3)^. This understanding underscores that the use of data from deceased persons should take into account not only potential social benefits but, above all, the moral duty to respect the individual’s autonomy and *ante-mortem* wishes.

In Brazil, current legislation-including Resolution No. 466/2012 of the National Health Council (Conselho Nacional de Saúde, CNS), the Brazilian General Data Protection Law (Lei Geral de Proteção de Dados, LGPD - Law No. 13,709/2018), and the recent Law No. 14,874/2024, which has not yet been regulated-establishes guidelines for the use of sensitive information and governs research involving human participants^(4-6)^.

These regulations are grounded in major international documents-such as the Declaration of Helsinki, the International Covenant on Civil and Political Rights, and the Universal Declaration on Bioethics and Human Rights-which reaffirm respect for human dignity at all stages of life^(7-9)^. However, the way these norms are applied to deceased patients still leaves significant gaps, particularly regarding the *post-mortem* protection of their data.

Internationally, the European Union provides general guidelines for the processing of personal data of its citizens. The General Data Protection Regulation, however, allows each country to legislate autonomously on specific aspects, such as *post-mortem* data protection^(10,11)^. In the United States, information from deceased patients can be used for up to 50 years after their death^(12,13)^.

There is, therefore, a need to examine the existing gaps, the ethical implications from a deontological standpoint, and possible regulatory solutions to this problem. Against this backdrop, reflection at both national and international levels is essential in the search for more specific and ethically sound legislation. Such efforts are crucial to ensure the responsible use of deceased patients’ data in line with the principles of dignity, autonomy, and privacy, and to foster trust in scientific research and in the protection of human rights.

In this context, the choice of Kantian deontology as the theoretical framework is justified because Kantian ethics establishes human dignity as an absolute value and autonomy as an inviolable principle, providing a robust foundation for analyzing the bioethical dilemmas associated with the *post-mortem* use of personal data. In contrast to consequentialist or utilitarian approaches, Kantian deontology requires that individuals always be treated as ends in themselves, thereby allowing reflection on ethical limits that persist even after death.

## OBJECTIVES

To examine the bioethical aspects of storing and retrieving deceased patients’ data for scientific research from a Kantian deontological perspective.

## METHODS

This theoretical-reflective essay examines the use of deceased patients’ data in scientific research, in light of current national^(4-6,8,14-16)^ and internationa^(7,9-13,17-26)^ legislation and a Kantian deontological approach.

Immanuel Kant’s deontological ethics holds that human dignity is an absolute and unconditional value that must be respected under all circumstances, including after death. According to this approach, individuals must always be treated as ends in themselves and never merely as means, which imposes ethical limits on the use of personal data even after death^(2,3)^.

The findings, explanations, and reflections are organized around thematic axes developed from interpretations of the existing literature and reflective insights informed by the adopted theoretical framework. The analysis is structured in three parts. First, we provide a historical and philosophical overview of death and human dignity. Second, we discuss national and international normative frameworks governing the use of deceased patients’ data. Finally, we offer a critical reflection grounded in the context of *post-mortem* organ donation for transplantation.

No prior ethical review was required, as this theoretical-reflective essay involves no direct interaction with human participants and no laboratory experimentation.

## RESULTS AND DISCUSSION

### Historical aspects of bioethics and its relationship to death

Death is a complex phenomenon whose meanings extend far beyond the biological domain. Different cultures and traditions ascribe their own meanings to dying, seeing it as a transition, rupture, return, or transformation. In contemporary societies, which are strongly shaped by scientific and technological rationality, dying has gradually been institutionalized, displaced from the family sphere to the hospital setting, and at times turned into a taboo. This shift directly affects how death is experienced and understood and whether it is respected, ethically and legally^(27-29)^.

Against this backdrop, bioethics emerges as a response to tensions between technological advances and fundamental human values. The term combines “bio,” from the Greek *bíos* (life), and “ethics,” from the Greek *ethikós* (relating to character or morals), and points to the need to discuss the moral implications of practices that affect life, particularly concerning human dignity and respect for life and death^(27)^.

Accounts of the emergence of bioethics often focus on the period after World War II. Some authors, however, describe its origins in terms of broad categories: technology-driven, issue-based, event-centered, institution-based, and rooted in gradual, multicausal growth. This plurality of perspectives on the rise of bioethics is often taken as part of the field’s foundation and helps explain its subsequent development^(30)^.

In Brazil, the history of bioethics began to gain prominence amid international developments, advances in the health sciences, and growing concern about ethical implications. In the United States and Europe, the field was already gaining momentum, especially after key events that sparked debates on human dignity, patient rights, and ethics in scientific research. In Brazil, these issues gradually entered the agenda of medical and academic institutions^(30)^.

The first bioethics debates in Brazil were shaped by the work of foreign scholars, by cases involving organ transplantation, and by the introduction of new health technologies that raised questions about the need for ethical norms and principles to guide these practices. In 1995, Brazil took an important step with the creation of the Brazilian Society of Bioethics (*Sociedade Brasileira de Bioética*, SBB), an organization devoted to promoting the study and debate of bioethical issues in the country^(30)^.

The advancement of bioethics in Brazil was also closely linked to the strengthening of ethical regulations governing scientific research involving human participants. A major milestone was Resolution No. 196/1996 of the National Health Council (*Conselho Nacional de Saúde*, CNS), which set out ethical guidelines for research involving human beings and established the Brazilian Research Ethics Committee system (CEP/CONEP system), comprising local Research Ethics Committees - RECs (*Comitês de Ética em Pesquisa*, CEPs) and the National Research Ethics Commission (*Comissão Nacional de Ética em Pesquisa*, CONEP)^(14)^.

In 2012, Resolution No. 196 was replaced by Resolution No. 466, which expanded and refined ethical criteria for such research, underscoring the importance of informed consent and the protection of vulnerable groups. Over time, the concept of bioethics broadened and increasingly focused on ethical issues related to health and scientific research, such as respect for human dignity, patient autonomy, informed consent, and justice in the allocation of health resources^(4)^.

From the early 2000s onward, debate within bioethics on end-of-life issues became more prominent, bringing to the forefront topics such as palliative care, orthothanasia, advance directives, and respect for the dignity of terminally ill patients. These discussions showed that end-of-life decisions require not only clinical judgment but also moral reflection on each person’s suffering, autonomy, and legacy^(27,28)^.

Bioethics seeks to balance respect for human dignity with the relief of suffering by taking into account principles such as patient autonomy, beneficence, and nonmaleficence in the care and services provided, the sanctity of life, and justice and equity in access to health care^(27)^.

In this regard, Kantian deontology provides a moral grounding for dignity after death, often referred to in Latin as *post mortem*, understood as “events, conditions, or measures that occur or take effect after the death of a person”^(31)^. For Kant, human dignity is an absolute value that does not depend on an individual’s usefulness or biological condition^(2,3)^. Even after death, the person must be treated as an end in themselves, which includes respect for their memory, their wishes expressed while alive, and the confidentiality of their information. The way the dead are treated, including in the context of scientific research, therefore says a great deal about a society’s ethical commitments.

Legal and regulatory aspects of key laws

Human dignity is a universal principle enshrined in numerous treaties and legal instruments. Proclaimed in 1948, the Universal Declaration of Human Rights (UDHR) states in Article 1 that “all human beings are born free and equal in dignity and rights” and affirms in Article 12 that “no one shall be subjected to arbitrary interference with his privacy […] nor to attacks upon his honour and reputation”^(9)^.

Although historically directed toward the living, these ethical precepts provide a foundation for reflecting on the protection of deceased persons’ data, since their memory, social identity, and dignity do not vanish with death.

Within Kant’s deontological ethics, dignity is understood as an intrinsic and unconditional value, which means that people must always be treated as ends in themselves and never merely as means to someone else’s purposes. From this standpoint, the same logic applies after death when sensitive information about deceased patients is at stake: such data cannot be reduced to instruments for scientific progress without regard for the autonomy previously exercised by the individual or for the memory they leave behind in society^(2,3)^. *Post-mortem* respect thus becomes an expression of Kantian dignity, which does not cease with the biological end of life.

In Brazil, the regulatory framework for research involving human participants has gradually developed. National Health Council Resolution No. 196/1996 was an early milestone, establishing guidelines for the ethical use of data in scientific research^(14)^. Key aspects include informed consent, the protection of vulnerable groups, and data confidentiality, all of which echo Kant’s categorical imperative by requiring that the person be recognized as a moral subject capable of deliberating about the use of their information.

Resolution No. 466/2012, which revoked the earlier regulation, expanded these provisions by more clearly establishing the requirement for an Informed Consent Form (ICF), emphasizing that ethical data handling depends on respecting the participant’s freely expressed wishes or those of their legal representative. The only exception to this rule, allowed solely in duly justified cases of public interest, must be submitted for review by the CEP/CONEP system to ensure that decisions are not made about the data of individuals who have no voice^(4)^. This demand for moral and institutional justification is consistent with deontological reasoning, since an action is morally valid only if it can be universalized and respects humanity in every person.

Despite these advances, significant gaps remain. By establishing the National System of Ethics in Research with Human Participants, Law No. 14,874/2024 represents a new step in consolidating the legal framework for research ethics in Brazil; however, its regulation through decrees and complementary ordinances is still underway and has left practical gaps, especially regarding the use of *post-mortem* data^(6)^. From a deontological standpoint, this lack of definition weakens the State’s moral duty to protect rights even after death, as it leaves room for unequal decisions and subjective interpretations.

The LGPD (Law No. 13,709/2018) also sets out guidelines for the processing of personal data, including for research purposes. Although it does not explicitly address data from deceased persons, the LGPD requires anonymization, consent, and legitimate purposes, elements that resonate with Kantian principles of autonomy and ethical rationality^(5)^. However, the absence of provisions specifically addressing *post-mortem* data weakens the full protection of these data subjects and shifts to RECs the difficult task of interpreting legal gaps under intense institutional pressures.

A Kantian reading of these regulatory gaps shows that they amount not only to a legal vacuum but also to an ethical shortcoming. By leaving the handling of deceased patients’ data undefined, there is a risk of reducing the person to a means for others’ interests, in conflict with Kant’s categorical imperative, according to which the moral value of actions lies not in their consequences but in the duty that guides them^(2,3)^. Thus, the lack of specific regulation for *post-mortem* data exposes an underlying tension between scientific progress and the preservation of human dignity.

Within RECs, the Commitment Form for the Use of Databases (*Termo de Compromisso para Utilização de Bancos de Dados*, TCUD) has become a practical tool, even though it lacks explicit legal backing. It seeks to ensure the ethical use of data from secondary sources, including records from deceased patients, grounded in anonymization, traceability, and a commitment to confidentiality^(32)^. However, the routine adoption of the TCUD without formal regulation reveals, from a Kantian deontological perspective, a regulatory weakness whereby what should be an exception-the use of sensitive information without direct consent-has become standard practice in some institutions.

The international landscape offers valuable points of comparison. The international landscape offers valuable points of comparison. In the United States, the Health Insurance Portability and Accountability Act (HIPAA permits the use of patients’ health information for up to 50 years after death for specific purposes such as research and teaching. Such use must remain limited and conditional on minimizing risks and preserving confidentiality, in recognition that death does not entirely extinguish the ethical ties to an individual’s data^(12,13)^.

In Europe, although the General Data Protection Regulation (GDPR) does not apply directly to deceased persons’ data, it allows Member States to legislate on this issue. Countries such as France, Italy, Portugal, Spain, and Estonia, among others, have already adopted specific provisions^(10,11,34,35)^. In France, for example, the Code Civil allows heirs to decide how the deceased person’s information will be used^(17)^.

In Catalonia, in northeastern Spain, Law 10/2017 recognized the possibility of expressing “digital wishes” during one’s lifetime, allowing individuals to determine the fate of their information after death. However, the creation of an electronic registry of these wishes was declared unconstitutional by the Spanish Constitutional Court, leading to its suspension. As a result, these provisions remain valid only when they are formalized in a will^(18,19,29)^. In Portugal, Law No. 58/2019 ensures that *post-mortem* data may be accessed by persons designated by the deceased, except when there is an explicit prohibition^(20)^.

In Australia, the federal Privacy Act 1988 does not recognize legal protection for *post-mortem* data and defines an “individual” solely as a living person. State laws such as the Health Records Act 2002 (New South Wales) and the Health Records Act 2001 (Victoria), however, extend the protection of deceased persons’ health information for up to 30 years and allow authorized legal representatives to decide on its use, subject to ethical criteria^(21-23)^.

In South Africa, the Protection of Personal Information Act (POPIA, 2013) applies exclusively to living individuals, and the protection of deceased persons’ data falls outside its scope^(24)^. In Rwanda, in East Africa, the Data Protection and Privacy Law (Law No. 058/2021) is one of the few on the African continent to explicitly include deceased individuals’ data within the scope of legal protection, although its regulation remains limited^(25)^.

In Japan, the Act on the Protection of Personal Information (APPI) covers only “personal information of living individuals” and does not extend to the deceased^(26)^.

These international models point to a principle that Kantian deontology has long emphasized: respect for a person’s will should be preserved even after death because dignity is a value that does not end with physical life. An individual’s ethical autonomy extends to the legacy they leave behind, including how their personal data may be used^(2,3)^.

The absence of explicit regulation in Brazil is therefore not only a legal issue but also a moral one. Without clear rules, sensitive information about deceased persons may be used in unequal, arbitrary, and potentially disrespectful ways, and may also undermine the ethical conduct of scientific research. From a Kantian standpoint, such uses instrumentalize the deceased person by treating them as a means to others’ ends, in tension with the moral duty to respect them as an end in themselves.

### Brain death, organ donation for transplantation, and the use of secondary medical record data for scientific research

This section does not aim to present an empirical example but to apply the discussion to the real and complex context of organ donation, fostering critical reflection on the ethical dilemmas surrounding the use of *post-mortem* data.

Brain death is a medical and legal milestone that marks the end of life, even when vital functions are artificially maintained through technological support. From that point onward, Brazilian law allows organ donation for transplantation, provided the family consents. However, this scenario is not confined to the biological dimension; it has far-reaching ethical implications, especially regarding the potential use of the deceased patient’s clinical information for scientific purposes^(1,35)^.

In this context, bioethics calls for a careful examination of principles such as autonomy, beneficence, and nonmaleficence. Brazilian legislation still does not explicitly regulate the use of information from deceased donors for research, and Research Ethics Committees must assess each case individually, often relying on instruments such as the Commitment Form for the Use of Databases^(6,15,16)^.

At present, consent for the use of information after death usually falls to family members. In addition to being bureaucratic, this process places a significant emotional burden on those grieving. Expecting emotionally vulnerable people to make complex, morally sensitive decisions within a short period of time may represent not only an additional strain but also an ethical injustice. In other words, responsibility that perhaps should have been institutionalized and/or decided by the individual while still alive is shifted onto bereaved relatives^(28,29)^.

Conversely, assigning this authorization solely to health institutions, even when data are properly anonymized, would raise serious questions about the deceased person’s autonomy and respect for their wishes. This is precisely where Kantian ethics comes to the fore. According to Kant, respect for a person’s dignity requires that they never be treated merely as a means to someone else’s ends, even when those ends are noble, such as scientific advancement, and even when these normative requirements are not applied universally to similar situations^(2,3)^. Therefore, using personal information without prior consent may violate this fundamental principle of deontological morality.

This tension becomes even more evident when contrasted with a utilitarian perspective. Organ donation, for instance, is often defended with utilitarian arguments in which “the ends justify the means”: using the body or its data is deemed acceptable as long as the collective benefit-such as saving lives-outweighs any potential discomfort or harm to the individua^(2,36)^. Within this view, the weight given to family consent, or its absence, may be downplayed as long as the greater good is achieved.

Kantian deontology does not accept this kind of moral flexibility. For Kant, the moral value of an action lies not in its consequences but in the intention and in the fulfillment of duty. This implies that, even when it brings benefits for science and society, the use of personal data may still be ethically inadmissible if it does not respect the person’s will^(2,3)^. The criterion is not the final balance of outcomes but, as noted earlier, whether the action could be universalized as a moral principle-that is, whether we would accept having our own information used without our consent, even after death.

Faced with this impasse, some international experiences, such as the implementation of advance directives in European countries, may serve as a source of inspiration. This is illustrated by the examples of Portugal and Catalonia, where legal mechanisms allow individuals, while still alive, to determine how their data and their digital identity will be handled after death^(18-20)^. Such strategies reinforce individual autonomy and lessen the burden of decision-making for family members or institutions.

From a Kantian deontological standpoint, the most ethical path may be precisely to allow people, while still alive and fully capable of making decisions, to express their wishes regarding the *post-mortem* use of their data. Such *ante-mortem* consent would ensure that the deceased are treated as ends in themselves, even after death, and would safeguard the moral principles that underpin a genuinely ethical society.

## FINAIS CONSIDERATIONS

The use of deceased patients’ data for scientific purposes should not be prohibited but rather carefully regulated. The debate is not about halting scientific progress but about building an ethical, legal, and human-centered framework to guide the responsible use of these data. The still incipient and unclear treatment of the *post-mortem* use of personal data in Brazilian regulations, especially in research settings, reveals a troubling gap.

The findings discussed in this article show that, although human dignity is widely recognized in national and international regulations, its effective protection after death still faces practical barriers. Individual autonomy, a central value in Kantian ethics, should not end with biological death. On the contrary, as Kantian deontology suggests, respect for the individual as an end in themselves requires that their wishes and personal data be treated with the same seriousness they command during life. Placing the near-exclusive responsibility for authorizing the use of such information on family members at a time of grief and emotional vulnerability imposes an unfair burden.

Conversely, entrusting this authorization entirely to institutions may violate the principle of informed consent. It is therefore reasonable to propose the creation of an *ante-mortem* consent instrument, inspired by the international models discussed, that would allow individuals to state in advance how they wish their data to be used after death. Such a measure reinforces the Kantian paradigm and helps ensure that autonomy and dignity remain central pillars, even after life has ended.

Thus, the reflection presented in this article not only exposes existing weaknesses but also points to possible ways of overcoming this impasse, underscoring the need for clear legislation, robust consent mechanisms, clearly defined institutional responsibilities, and ethics education.

Science must, of course, continue to advance, but it cannot lose sight of its central focus: the human person, whether alive or deceased. From a Kantian perspective, building such normative instruments is more than a technical or administrative task; it is a moral duty. The categorical imperative demands that respect for humanity be universal. This means that if we regard the use of our own data without consent after death as unacceptable, we must, for the sake of consistency, reject this practice in all cases. Information about deceased persons should not be treated as neutral traces of data but recognized as an extension of the person who lived.

## Data Availability

Not applicable.
