# Supplementary material for: Storage and retrieval of deceased patients’ data: a bioethical reflection from a Kantian deontological perspective
Source: Rev Bras Enferm. 2026 Jul 24;79:e20250302. doi: 10.1590/0034-7167-2025-0302 (PMC13403632; doi:10.1590/0034-7167-2025-0302)
Supplement: Translation

# Translation: Armazenamento e recuperação de informações de pacientes falecidos: reflexão bioética à luz da deontologia kantiana

**DOI:** 10.1590/0034-7167-2025-0302pt

**Authors:** Bethânia Karoline Alvaro Menezes, Marcos Antonio Ferreira, Aline Moraes Silva, Andreia Insabralde Queiroz-Cardoso, Viviane Euzébia Pereira Santos

**Affiliations:** I Universidade Federal de Mato Grosso do Sul. Campo Grande, Mato Grosso do Sul, Brasil; II Universidade Federal do Rio Grande do Norte. Natal, Rio Grande do Norte, Brasil

**Keywords:** Armazenamento e Recuperação da Informação, Morte, Teoria Ética, Bioética, Reflexão.

## Abstract

**Objetivos:**

promover reflexão sobre os aspectos bioéticos no armazenamento e recuperação de informações de pacientes falecidos para pesquisa científica à luz da deontologia kantiana.

**Métodos:**

estudo teórico-reflexivo fundamentado na ética deontológica kantiana e em legislações nacionais e internacionais.

**Resultados:**

observam-se fragilidades na regulamentação do uso de informações de pacientes falecidos no Brasil. Normas nacionais de ética em pesquisa e proteção de dados não abordam claramente a temática, enquanto modelos do cenário internacional apontam para diferentes soluções relacionadas à vontade do paciente após a morte. A deontologia kantiana destaca a dignidade como um valor absoluto que persiste além da vida biológica e exige respeito à autonomia e à vontade previamente expressa.

**Considerações Finais:**

Sugere-se a criação de instrumentos legais de consentimento em vida que permitam ao paciente decidir sobre o uso de seus dados após sua morte, visando garantir sua dignidade e autonomia.

## INTRODUÇÃO

A morte é compreendida de diversas maneiras em diferentes culturas e religiões; o “morrer” biológico constitui sequências de fenômenos gradativos^(1)^, e não um momento único e finito. Essa compreensão traz implicações práticas, especialmente no contexto de pesquisas científicas que usam informações de pacientes após a morte.

O avanço da pesquisa em saúde depende cada vez mais do acesso a informações detalhadas e precisas, que frequentemente incluem dados sensíveis de pacientes falecidos. A coleta, o armazenamento e a análise dessas informações envolvem questões éticas relevantes, já que, sob a perspectiva deontológica kantiana, o respeito à dignidade humana é um valor absoluto que não se extingue com a morte^(2,3)^. Esse entendimento reforça que o uso de informações de pessoas falecidas deve considerar não apenas os potenciais benefícios sociais, mas sobretudo o dever moral de respeitar a autonomia e a vontade/desejo do indivíduo.

No Brasil, a legislação vigente - composta pela Resolução nº 466/2012 do Conselho Nacional de Saúde (CNS), pela Lei Geral de Proteção de Dados (LGPD - nº 13.709/2018) e pela recente Lei nº 14.874/2024, ainda não regulamentada - estabelece diretrizes sobre o uso de informações sensíveis e regulam as pesquisas com seres humanos^(4-6)^.

Essas normativas encontram respaldo em importantes documentos internacionais - como a Declaração de Helsinque; o Pacto Internacional sobre os Direitos Civis e Político; e a Declaração Universal sobre Bioética e Direitos Humanos - que reforçam o respeito à dignidade humana em todas as fases da vida^(7-9)^. Entretanto, a aplicação dessas normas em relação a pacientes falecidos ainda apresenta lacunas, especialmente no que tange à proteção de seus dados após sua morte.

No cenário internacional, a União Europeia fornece diretrizes gerais para o uso de informações pessoais de cidadãos dos Estados-membros. No entanto, o regulamento permite que cada país legisle autonomamente sobre aspectos específicos, como a proteção de dados *post mortem* ^(10,11)^. Já nos Estados Unidos da América, informações de pacientes falecidos podem ser utilizadas por até 50 anos após sua morte^(12,13)^.

Portanto, é necessário promover uma reflexão sobre as lacunas existentes, implicações éticas à luz da deontologia e possíveis soluções normativas para a problemática apresentada. Diante desse panorama, torna-se imprescindível uma reflexão em níveis nacional e internacional em busca de uma legislação mais específica e ética. Tal esforço é fundamental para assegurar que o uso de dados de pacientes falecidos seja realizado de forma responsável, com respeito aos princípios da dignidade, autonomia e privacidade, além de promover a confiança na pesquisa científica e na proteção dos direitos humanos.

Nesse sentido, a escolha da deontologia kantiana como referencial teórico justifica-se pelo fato de que a ética kantiana, ao estabelecer a dignidade humana como valor absoluto e a autonomia como princípio irrenunciável, oferece um arcabouço sólido para analisar os dilemas bioéticos relacionados ao uso de dados *post mortem*. Em oposição a correntes consequencialistas ou utilitaristas, a deontologia kantiana exige que os indivíduos sejam tratados sempre como fins em si mesmos, o que possibilita refletir sobre limites éticos mesmo após a morte.

## OBJETIVOS

Promover uma reflexão sobre os aspectos bioéticos no armazenamento e recuperação de informações de pacientes falecidos para fins de pesquisa científica à luz da deontologia kantiana.

## MÉTODOS

Estudo de ensaio teórico-reflexivo realizado no contexto da pesquisa científica que envolve uso de informações de pacientes falecidos, à luz das legislações nacionais^(4-6,8,14-16)^ e internacionais vigentes^(7,9-13,17-26)^ e da abordagem deontológica kantiana.

A ética deontológica de Immanuel Kant estabelece que a dignidade humana é um valor absoluto e incondicional a ser respeitado em todas as circunstâncias, inclusive após a morte. Essa abordagem sustenta que os indivíduos devem ser tratados sempre como fins em si mesmos e não como meios, o que impõe limites éticos para o uso de informações pessoais, mesmo após seu falecimento^(2,3)^.

A apresentação dos resultados encontrados e das explanações e reflexões desenvolvidas neste estudo segue os eixos temáticos construídos com base em interpretações da literatura existente e de impressões reflexivas à luz do referencial teórico adotado. A análise foi organizada em três partes. Inicialmente, se apresenta uma contextualização histórica e filosófica sobre a morte e dignidade humana. Em seguida, discutem-se os marcos normativos nacionais e internacionais que regulam o uso de informações de pacientes falecidos. Por fim, propõe-se uma reflexão crítica fundamentada na análise do contexto da doação de órgãos para transplantes.

Cabe destacar que a apreciação ética prévia foi dispensada, por se tratar de um estudo de ensaio teórico-reflexivo, sem interação direta com seres humanos e sem experimentação laboratorial.

## RESULTADOS E DISCUSSÃO

### Aspectos históricos da bioética e sua relação com a morte

A morte é um fenômeno complexo, com significados que ultrapassam o âmbito biológico. Diferentes culturas e tradições atribuem sentidos próprios ao morrer, compreendendo-o como transição, ruptura, retorno ou transformação. Na sociedade contemporânea, fortemente marcada pela racionalidade técnico-científica, o morrer foi progressivamente institucionalizado, deslocado do espaço familiar para o hospitalar e, por vezes, silenciado como tabu. Esse deslocamento impacta diretamente a forma como a morte é vivida e compreendida, além do modo como ela é respeitada, ou não, tanto do ponto de vista ético quanto jurídico^(27-29)^.

É nesse contexto que se estabelece a bioética como uma resposta às tensões entre os avanços tecnológicos e os valores humanos fundamentais. O termo resulta da junção entre “bio”, do grego *bíos* (vida), e “ética”, do grego *ethikós* (relativo ao caráter ou moral); e surge da necessidade de discutir as implicações morais das práticas relacionadas à vida, particularmente no tocante à dignidade humana e ao respeito pela vida e pela morte^(27)^.

O surgimento da bioética se relaciona ao período posterior à Segunda Guerra Mundial. No entanto, há um olhar sobre sua origem que permite dividi-la em diferentes categorias gerais: impulsionada pela tecnologia; baseada em temas; centrada em eventos; apoiada na institucionalização; e enraizada no crescimento gradual e multicausal. Essa diversidade de visões referentes à eclosão da bioética pode ser vista como um fator de fundamentação para seu crescimento e desenvolvimento por aqueles que relatam essa história^(30)^.

Impulsionado pelo cenário internacional, avanços das ciências da saúde e crescente preocupação com as implicações éticas, a história da bioética no Brasil começou a ganhar relevância. O movimento já estava em ascensão com as discussões sobre a dignidade humana, proteção dos direitos dos pacientes e ética em pesquisas científicas, principalmente nos Estados Unidos e na Europa, após adventos importantes. Já no Brasil, essas questões começaram a ser discutidas em instituições médicas e acadêmicas^(30)^.

As primeiras discussões sobre bioética no Brasil foram influenciadas por obras de autores estrangeiros, pelo surgimento de casos envolvendo transplantes de órgãos e pela introdução de novas tecnologias em saúde que provocaram reflexões sobre a necessidade de normas e princípios éticos que orientassem tais práticas. Em 1995, o Brasil deu um passo importante com a criação da Sociedade Brasileira de Bioética (SBB), instituição responsável por promover o estudo, a pesquisa e o debate sobre temas bioéticos no país^(30)^.

O avanço da bioética no Brasil também relacionou-se ao fortalecimento das normativas éticas para a condução de pesquisas científicas que envolvessem seres humanos. A primeira regulamentação de destaque foi a Resolução nº 196/1996 do CNS, que estabeleceu diretrizes éticas para pesquisas com seres humanos e criação do sistema CEP/CONEP, dos Comitês de Ética em Pesquisa (CEP) e da Comissão Nacional de Ética em Pesquisa (CONEP)^(14)^.

Em 2012, a Resolução nº 196 foi substituída pela Resolução nº 466, que ampliou e detalhou os critérios éticos para a realização de pesquisas, com destaque para a importância do consentimento livre e esclarecido e da proteção de grupos vulneráveis. O conceito de bioética ampliou-se ao longo do tempo e passou a focar mais nas questões éticas relacionadas à saúde e à pesquisa científica, como o respeito à dignidade humana, a autonomia do paciente, o consentimento informado e a justiça na distribuição de recursos em saúde^(4)^.

A partir da primeira década dos anos 2000, ampliou-se o debate bioético em torno do final da vida, ressaltando-se temas como cuidados paliativos, ortotanásia, diretivas antecipadas de vontade e respeito à dignidade do paciente em fase terminal. Tais discussões evidenciaram que o fim da vida exige não apenas decisões clínicas, mas também ponderações morais sobre o sofrimento, autonomia e legado de cada indivíduo^(27,28)^.

A bioética busca equilibrar o respeito pela dignidade humana com o alívio do sofrimento, ao levar em consideração princípios como a autonomia do paciente; a beneficência e não maleficência das assistências e serviços prestados; a sacralidade pelo respeito à vida; e a justiça e equidade no acesso aos cuidados de saúde^(27)^.

Nesse sentido, a ética deontológica kantiana contribui com a fundamentação moral da dignidade após a morte (do latim, *post mortem*), que se refere a “eventos, condições ou medidas que ocorrem ou têm efeito após a morte de uma pessoa”^(31)^. Para Kant, a dignidade humana trata de um valor absoluto que independe da utilidade ou da condição biológica do sujeito^(2,3)^. Mesmo após a morte, o indivíduo deve ser tratado como um fim em si, o que inclui o respeito à sua memória, aos seus desejos expressos em vida e à confidencialidade de suas informações. Assim, a forma como são tratados os mortos, inclusive no âmbito da pesquisa científica, revela muito da ética sustentada pela sociedade.

### Aspectos legais e normativos das principais leis

A dignidade humana é um princípio universal reconhecido em diversos tratados e legislações. Proclamada em 1948, a Declaração Universal dos Direitos Humanos (DUDH) estabelece, em seu Artigo 1º, que “todos os seres humanos nascem livres e iguais em dignidade e em direitos”; e afirma, no Artigo 12, que “ninguém será sujeito a interferência arbitrária na sua vida privada […] nem a ataques à sua honra e reputação”^(9)^.

Embora historicamente direcionados aos vivos, esses preceitos éticos fundamentam a reflexão sobre a proteção de informações de pessoas falecidas, cuja memória, identidade social e dignidade não deixam de existir após a morte.

No centro da ética deontológica de Kant, a dignidade é compreendida como valor intrínseco e incondicional, ou seja, as pessoas devem ser tratadas sempre como fins em si mesmas e nunca como meios para fins alheios. Nesse sentido, mesmo após a morte, essa lógica se sustenta ao lidar com informações sensíveis de pacientes falecidos: não se pode reduzi-las a instrumentos em prol do avanço científico sem considerar a autonomia previamente exercida pelo indivíduo ou a memória que ele deixa na sociedade^(2,3)^. Portanto, o respeito *post mortem* é a expressão da dignidade kantiana, ou seja, ele não se extingue com o fim biológico da vida.

No Brasil, o arcabouço normativo relacionado à pesquisa com seres humanos tem sido construído gradualmente. A Resolução nº 196/1996 do CNS foi um marco inicial, ao estabelecer diretrizes para o uso ético de informações em pesquisas científicas^(14)^. A preocupação com o consentimento informado, a proteção de grupos vulneráveis e a confidencialidade dos dados são aspectos fundamentais que dialogam com o imperativo categórico kantiano, na medida em que exigem o reconhecimento da pessoa enquanto sujeito moral, capaz de deliberar sobre o uso de suas informações.

A Resolução nº 466/2012, que revogou a anterior, ampliou essas disposições ao estabelecer, mais claramente, a obrigatoriedade do Termo de Consentimento Livre e Esclarecido (TCLE), reforçando que o tratamento ético dos dados exige o respeito à vontade livremente manifestada pelo participante ou seu representante legal. A exceção a essa regra, permitida apenas em casos justificados de interesse público, deve ser submetida à avaliação do sistema CEP/CONEP, para garantir que a decisão não recaia sobre os dados de indivíduos sem voz^(4)^. Essa exigência de justificação moral e institucional ressoa com a lógica deontológica, pois uma ação só é moralmente válida se puder ser universalizada e respeitar a humanidade no outro.

Apesar dos avanços, ainda há lacunas importantes. Ao instituir o Sistema Nacional de Ética em Pesquisa com Seres Humanos, a Lei nº 14.874/2024 representa um novo passo na consolidação do marco legal da ética em pesquisa, mas sua regulamentação por decretos e portarias complementares ainda está em curso e apresenta lacunas práticas, especialmente no que tange ao uso de informações *post mortem* ^(6)^. Sob a ótica deontológica, essa indefinição enfraquece o dever moral do Estado de proteger os direitos mesmo após a morte, ao deixar o campo aberto para decisões desiguais e interpretações subjetivas.

A LGPD nº 13.709/2018 também contribui com diretrizes para o tratamento de informações pessoais, inclusive em pesquisas. Embora não trate explicitamente de dados de pessoas falecidas, a LGPD estabelece a necessidade de anonimização, consentimento e finalidades legítimas, elementos que dialogam com os princípios kantianos da autonomia e da racionalidade ética^(5)^. Entretanto, a ausência de disposições específicas para dados *post mortem* enfraquece a proteção plena desses sujeitos e transfere aos CEPs a difícil tarefa de interpretar lacunas jurídicas sob fortes pressões institucionais.

A leitura kantiana dessas lacunas normativas evidencia que não se trata apenas de um vazio jurídico, mas também de uma insuficiência ética. Ao deixar indefinido o tratamento dos dados de pacientes falecidos, corre-se o risco de reduzir a pessoa a um meio para interesses alheios, em desacordo com o imperativo categórico de Kant, em que o valor moral das ações não está nas consequências, mas no dever que as orienta^(2,3)^. Assim, a ausência de regulamentação específica para dados *post mortem* desvela um embate entre avanços científicos e a preservação da dignidade humana.

No contexto dos CEPs, o Termo de Compromisso para Utilização de Bancos de Dados (TCUD) constitui uma ferramenta prática, ainda que sem respaldo legal explícito. Ele busca garantir o uso ético de dados de fontes secundárias, inclusive de pacientes falecidos, com base em anonimização, rastreabilidade e compromisso com a confidencialidade^(32)^. No entanto, o uso do TCUD sem regulamentação oficial expõe, na visão deontológica kantiana, uma fragilidade normativa, na qual o que deveria ser exceção - o uso de informações sensíveis sem consentimento direto - tem se tornado uma prática sistemática em determinadas instituições.

O cenário internacional oferece contrapontos valiosos. No cenário internacional, destacam-se contrapontos valiosos. Nos Estados Unidos, a *Health Insurance Portability and Accountability Act* (HIPAA) permite o uso de informações de saúde de pacientes por até 50 anos após sua morte para fins específicos de pesquisa e ensino. O uso deve ser limitado e condicionado à minimização de riscos e à manutenção da confidencialidade, ao reconhecer que a morte não extingue totalmente os vínculos éticos com os dados do indivíduo^(12,13)^.

Na Europa, embora a Regulamentação Geral de Proteção de dados (do inglês, *General Data Protection Regulation* - GDPR) não se aplique diretamente a dados de pessoas falecidas, ela permite que os países legislem sobre o tema. França, Itália, Portugal, Espanha, Estônia, entre outros, já estabeleceram normativas específicas^(10,11,33,34)^. Na França, por exemplo, o *Code Civil* permite que os herdeiros decidam sobre o uso de informações do falecido^(17)^.

Já na Catalunha, região nordeste da Espanha, a Lei 10/2017 reconheceu a possibilidade de expressar “vontades digitais” em vida, permitindo que a pessoa defina o destino de suas informações após sua morte. Entretanto, a criação do Registro Eletrônico dessas vontades foi considerada inconstitucional pelo Tribunal Constitucional da Espanha, o que resultou na sua suspensão. Assim, as disposições continuam válidas apenas se estiverem formalizadas em testamento^(18,19,29)^. Em Portugal, a Lei nº 58/2019 assegura que dados *post mortem* possam ser acessados por pessoas designadas pelo falecido, exceto em caso de proibição expressa^(20)^.

Na Austrália, a *Privacy Act* 1988 (lei federal) não reconhece proteção legal a dados *post mortem*, e define o “indivíduo” apenas entre pessoas vivas. Contudo, leis estaduais como a *Health Records Act* 2002 (Nova Gales do Sul) e a *Health Records Act* 2001 (Victoria) estendem, por até 30 anos, a proteção das informações de saúde de pessoas falecidas e permitem decisão por representantes legais autorizados sob critérios éticos^(21-23)^.

Já na África do Sul, a *Protection of Personal Information Act* (POPIA, 2013) aplica-se exclusivamente a indivíduos vivos. A proteção dos dados de pessoas falecidas não está prevista nessa lei^(24)^. Na Ruanda, África Oriental, a *Data Protection and Privacy Law* (Lei nº 058/2021) é uma das raras no continente africano a incluir expressamente os dados de indivíduos falecidos como sujeitos de proteção legal, embora ainda com regulamentação limitada^(25)^.

No Japão, a *Act on the Protection of Personal Information* (APPI) cobre apenas “informações pessoais de indivíduos vivos” e não se estende aos falecidos^(26)^.

Esses modelos internacionais apontam o que a deontologia kantiana já antecipa como princípio: o respeito à vontade do sujeito deve ser preservado, mesmo após a sua morte, pois a dignidade é um valor que não se esgota com a vida física. A autonomia ética de um indivíduo se estende ao legado que deixa, inclusive no tocante ao uso de suas informações pessoais^(2,3)^.

A falta de regulamentação explícita no Brasil não é apenas um problema jurídico, mas um dilema moral. Sem regras claras, abre-se margem para que informações sensíveis de pessoas falecidas sejam utilizadas de forma desigual, arbitrária e potencialmente desrespeitosa, podendo infringir também a ética aplicada às pesquisas cientificas. Sob a ótica kantiana, esse tipo de uso instrumentaliza o sujeito falecido ao tratá-lo como meio para fins alheios, em descompasso com o dever moral de respeitá-lo na qualidade de um fim em si mesmo.

### A morte encefálica, a doação de órgãos para transplantes e a utilização de dados secundários de prontuários para fins de pesquisa científica

Este tópico não propõe apresentar um exemplo empírico, e sim aplicar a discussão à luz de um contexto real e complexo, o da doação de órgãos, visando fomentar uma reflexão crítica sobre os dilemas éticos que envolvem o uso de dados *post mortem*.

A morte encefálica é um marco médico e jurídico que define o fim da vida, ainda que haja manutenção artificial das funções vitais por meio de suporte tecnológico. A partir desse momento, é possível, nos termos da lei brasileira, realizar a doação de órgãos para fins de transplantes, desde que haja o consentimento familiar. No entanto, esse cenário não se restringe à questão biológica: desdobra-se em implicações éticas profundas, especialmente quando se cogita o uso das informações clínicas do paciente falecido para fins científicos^(1,35)^.

A bioética nesse contexto exige uma análise cuidadosa de princípios como a autonomia, a beneficência e a não maleficência. A legislação brasileira ainda não prevê explicitamente o uso de informações de doadores falecidos para pesquisa, e cabe aos CEPs avaliar caso a caso, muitas vezes com base em documentos como o TCUD^(6,15,16)^.

Atualmente, o consentimento para uso de informações após a morte recai geralmente sobre os familiares. Além de burocrático, esse processo impõe uma carga emocional significativa àqueles que estão em estado de luto. Esperar que pessoas fragilizadas tomem decisões complexas e moralmente sensíveis em um curto espaço de tempo pode representar não apenas um ônus emocional, mas também uma injustiça ética. Em outras palavras, transfere-se para os enlutados uma responsabilidade que talvez devesse ser previamente institucionalizada e/ou deliberada em vida pelo próprio sujeito^(28,29)^.

Por outro lado, delegar exclusivamente às instituições de saúde essa autorização, mesmo com a devida anonimização dos dados, levantaria questões sérias sobre a autonomia do falecido e respeito à sua vontade. É aqui que a ética kantiana se impõe com sua moralidade. Segundo Kant, a dignidade da pessoa exige que ela nunca seja tratada como um meio para fins alheios, mesmo que os fins sejam nobres, como o avanço da ciência ou ainda que essas exigências normativas não sejam aplicadas de forma universal a todas as situações semelhantes^(2,3)^. Portanto, o uso de informações pessoais sem consentimento prévio pode violar esse princípio fundamental da moralidade deontológica.

Essa tensão se torna ainda mais evidente quando contrastada com a ótica utilitarista. A doação de órgãos, por exemplo, é frequentemente defendida com base em argumentos utilitaristas, nos quais “os fins justificam os meios”, ou seja, justifica-se o uso do corpo ou de seus dados desde que o benefício coletivo, como salvar vidas, seja maior do que o possível desconforto ou dano individual^(2,36)^. Sob essa lógica, o consentimento familiar ou a ausência dele pode ser relativizado, contanto que o bem maior seja atingido.

A deontologia kantiana não aceita essa flexibilização moral. Para Kant, o valor moral de uma ação não está nas suas consequências, mas na intenção e no cumprimento do dever. Isso significa que, mesmo trazendo benefícios para a ciência e para a sociedade, o uso das informações ainda pode ser eticamente inadmissível se não respeitar a vontade do sujeito^(2,3)^. O critério não é o saldo final da ação, e sim, como referido anteriormente, se ela poderia ser universalizada como princípio moral, ou seja, se gostaríamos que nossas próprias informações fossem utilizadas sem nosso consentimento, mesmo após a morte.

Diante desse impasse, algumas experiências internacionais, como a implementação de diretivas antecipadas de vontade em países europeus, podem servir de inspiração. Essa questão é ilustrada pelos exemplos de Portugal e da Catalunha, onde existem mecanismos legais permitem ao cidadão declarar em vida o destino de seus dados e de sua identidade digital após a morte^(18-20)^. Tais estratégias reforçam a autonomia do indivíduo e reduzem o peso da decisão pelos familiares ou instituições.

Talvez o caminho mais ético, à luz da deontologia kantiana, seja exatamente o de permitir que a própria pessoa, enquanto ainda viva e em plena capacidade de decisão, manifeste sua vontade sobre o uso de seus dados *post mortem*. Esse consentimento antecipado garantiria o tratamento do falecido como um fim por “si mesmo”, mesmo após sua morte, e resguardaria os princípios morais que sustentam uma sociedade verdadeiramente ética.

## CONSIDERAÇÕES FINAIS

A utilização de informações de pacientes falecidos para fins científicos não deve ser proibida, mas, sim, cuidadosamente regulada. O debate não reside em impedir o avanço da ciência, mas em construir um alicerce ético, legal e humanizado que oriente o uso responsável desses dados. As informações pouco consolidadas e claras das normativas brasileiras sobre o uso *post mortem* de dados pessoais, especialmente no contexto da pesquisa, revela uma lacuna preocupante.

Os achados discutidos neste artigo evidenciam que, embora a dignidade do ser humano seja amplamente reconhecida nas normativas nacionais e internacionais, sua efetiva preservação após a morte ainda encontra barreiras práticas. A autonomia do sujeito, valor central na ética kantiana, não deveria cessar com o fim da vida biológica. Pelo contrário, como propõe o referencial deontológico, o respeito ao indivíduo enquanto fim em si mesmo exige que sua vontade e seus dados sejam tratados com a mesma seriedade de quando vivo. Atribuir aos familiares a responsabilidade quase exclusiva pela autorização do uso dessas informações em momentos de luto e vulnerabilidade emocional impõe uma carga injusta.

Por outro lado, confiar integralmente essa autorização às instituições pode violar o princípio do consentimento livre e esclarecido. Por isso, é válido propor a criação de um instrumento de consentimento em vida, a exemplo dos cenários internacionais apresentados, que permita aos indivíduos a expressão prévia de sua vontade quanto ao uso de seus dados após a morte. Essa medida reforça o paradigma kantiano e assegura que a autonomia e a dignidade do indivíduo permaneçam como pilares centrais, mesmo após o fim da vida.

Assim, a reflexão apresentada nesse artigo não apenas evidencia fragilidades existentes, mas também aponta vislumbres de possíveis caminhos para superar esse impasse, reforçando a necessidade de legislações claras, mecanismos de consentimento, responsabilidade institucional bem delimitada e educação ética.

Portanto, a ciência deve avançar, mas não pode se esquecer do seu foco principal: o ser humano, em vida ou não. Sob a ótica kantiana, a construção desses instrumentos normativos é mais que uma questão técnica ou administrativa: é um dever moral. O imperativo categórico orienta ser imprescindível que o respeito à humanidade seja universal. Isso implica que, uma vez sendo inaceitável o uso de dados próprios sem consentimento após a morte, deve-se, por coerência, rejeitar essa prática universalmente. Que as informações dos falecidos, em vez de serem tratadas como vestígios neutros de dados, sejam reconhecidas como uma extensão da pessoa que existiu.

## Data Availability

Não se aplica.
